# Efficacy of Glass Ionomer Cement as Pit and Fissure Sealant in Permanent First Molars

**DOI:** 10.7759/cureus.55882

**Published:** 2024-03-10

**Authors:** Amara Nazir, Humara Iqbal, Asad Mehmood, Muhammad A Khan, Zunaira Shaukat, Zaineb Abbas, Muhammad Kashif

**Affiliations:** 1 Operative Dentistry, Bakhtawar Amin Medical and Dental College, Multan, PAK; 2 Operative Dentistry, Multan Medical & Dental College, Multan, PAK; 3 Oral Biology, Lahore Medical and Dental College, Lahore, PAK; 4 Science of Dental Materials, Multan Medical & Dental College, Multan, PAK; 5 Oral Biology, Bakhtawar Amin Medical and Dental College, Multan, PAK; 6 Oral Pathology, Bakhtawar Amin Medical and Dental College, Multan, PAK

**Keywords:** sealants, pit and fissure, glass ionomer cement, dental composites, dental caries

## Abstract

Background/Objectives

Pit and fissure caries constitute a predominant portion, approximately 90% in permanent posterior teeth and 44% in primary teeth among children and adolescents. Among various preventive modalities, pit and fissure sealants play a pivotal role in safeguarding these vulnerable areas. Categorized by materials such as glass ionomer, composites, and polyacid-modified glass ionomers, these sealants offer effective protection. This study aims to evaluate the efficacy of glass ionomer-based pit and fissure sealants in terms of retention rate at 12-month post-procedure period in permanent first molars.

Methodology

This study was conducted at the Department of Operative Dentistry, Nishtar Institute of Dentistry, Multan, Pakistan. Fifty-six children, aged 7 to 12 years, presenting with pit and fissure caries in permanent first molar teeth were enrolled. Glass ionomer sealant was meticulously applied to the affected pits and fissures. The efficacy was assessed after 12 months based on predefined criteria.

Results

The age of participants ranged from 7 to 12 years, with a mean age of 9.24 ± 1.38 years. Among the 56 patients, 23 (41.2%) were male and 33 (58.8%) were female. Sealant retention was noted in 31 (55.35%) patients, while 25 (44.65%) experienced sealant loss. In the 7 to 9-year age group, 19 demonstrated complete sealant retention, whereas in the 10 to 12-year age group, 12 exhibited complete retention. Concerning gender distribution, 17 males and 14 females exhibited complete sealant retention.

Conclusion

Glass ionomer-based sealants demonstrate excellent properties for pit and fissure sealing owing to their low technique sensitivity, cost-effectiveness, and favorable retention rates. Therefore, they represent an optimal choice for this preventive dental procedure.

## Introduction

Dental caries, a complex disease, results in tooth demineralization by bacteria, the rate of which varies depending on the balance between demineralization and remineralization. This process leads to cavitation in enamel and dentin, potentially affecting the pulp [1]. The primary cause of caries is the production of lactic acid by cariogenic bacteria within the plaque biofilm, influenced by factors such as tooth susceptibility, dietary sugar intake, saliva composition, and duration of exposure [2]. During the 18th and 19th centuries, caries became widespread globally due to the easy accessibility of sugar to nearly every individual. Globally, caries prevalence ranges from 50 to 70 percent, with a specific study in 2021 reporting a prevalence rate of 60 percent in Pakistan [3].

Pit and fissure caries constitute approximately 90% of caries in permanent posterior teeth and 44% in primary teeth among children and teenagers [4]. The overall reduction in caries prevalence is largely attributed to the adoption of preventive measures like topical fluoride treatment, plaque management, community water fluoridation, and dietary sugar control [5]. These approaches have been notably successful in diminishing carious lesions on smooth surfaces. Pits and fissures are more prone to caries compared to smooth surfaces because they tend to retain plaque, posing challenges for effective cleaning and potentially impeding the protective benefits of fluoride [6].

Pit and fissure sealants stand out as highly effective methods essential for protecting vulnerable pits and fissures [7]. The application of sealants to these susceptible areas constitutes a conservative preventive technique against cavities. Subsequently, the sealant forms a micromechanical bond with the tooth, establishing a physical barrier that inhibits bacteria access to their nutrient source [8]. Despite the general rise in sealant utilization, they remain underutilized globally, despite extensive documentation in the literature showcasing their efficacy and caries-preventive benefits [9].

In contemporary dentistry, pit and fissure sealants are recognized as a comprehensive strategy for preventing caries at both the community and individual levels [10]. These sealants act as a physical barrier between microbes and the deep pit and fissure system, effectively halting the progression of caries on occlusal surfaces of healthy teeth, non-cavitated carious lesions, and incipient carious lesions [11].

Pit and fissure sealants are classified based on the materials used, including Glass Ionomer, Composites, and Polyacid-modified glass ionomers [12]. Glass Ionomer Cement (GIC) offers a viable option for the typically moist environment of the oral cavity. Due to its hydrophilic properties, GIC exhibits lower susceptibility to moisture compared to hydrophobic resin materials. GIC-based fissure sealants may demonstrate lower retention rates in pits and fissures compared to resin-based sealants [13]. However, even in cases where GIC sealants appear "partially" or "totally" lost clinically, trace amounts of material may still remain. The efficacy of GIC sealants is attributed to various factors such as the release of fluoride, the separation of bacteria from nutrients in the substrate beneath sealed early carious lesions, or a combination of these mechanisms [13].

While resin-based sealants are popular, they are often technique-sensitive and typically do not release fluoride ions, which could enhance protection against dental caries. GIC, specially formulated for fissure sealing, is gaining popularity due to its distinct advantages over resin-based sealants, including biocompatibility, chemical bonding with enamel and dentin, and fluoride ion release [14]. Unlike resin-based sealants, they are less susceptible to moisture contamination. Numerous studies have investigated the adhesion rate of fissure sealants, with poor retention and sealant loss being common issues [9]. Therefore, this study aims to assess the efficacy of GIC in terms of retention rate at 12-month post-procedure period after application in permanent molars.

## Materials and methods

Study setting and participants

This cross-sectional study was conducted at the Department of Operative Dentistry, Nishtar Institute of Dentistry, Multan, Pakistan, subsequent to approval from the Institutional Research Board (CPSP/REU/DSG-2016-102-1811). The study targeted children aged 7 to 12 years with sound/non-carious pits and fissures in their permanent first molar teeth. The sample size of 56 patients was determined with a 95% confidence level and a 5% margin of error.

Inclusion and exclusion criteria

Inclusion criteria comprised children aged 7 to 12 years with sound pits and fissures in their permanent first molar teeth. Exclusion criteria included children with physical or mental disabilities, known diabetes mellitus, or allergies to GIC.

Study procedure

**Figure 1:**
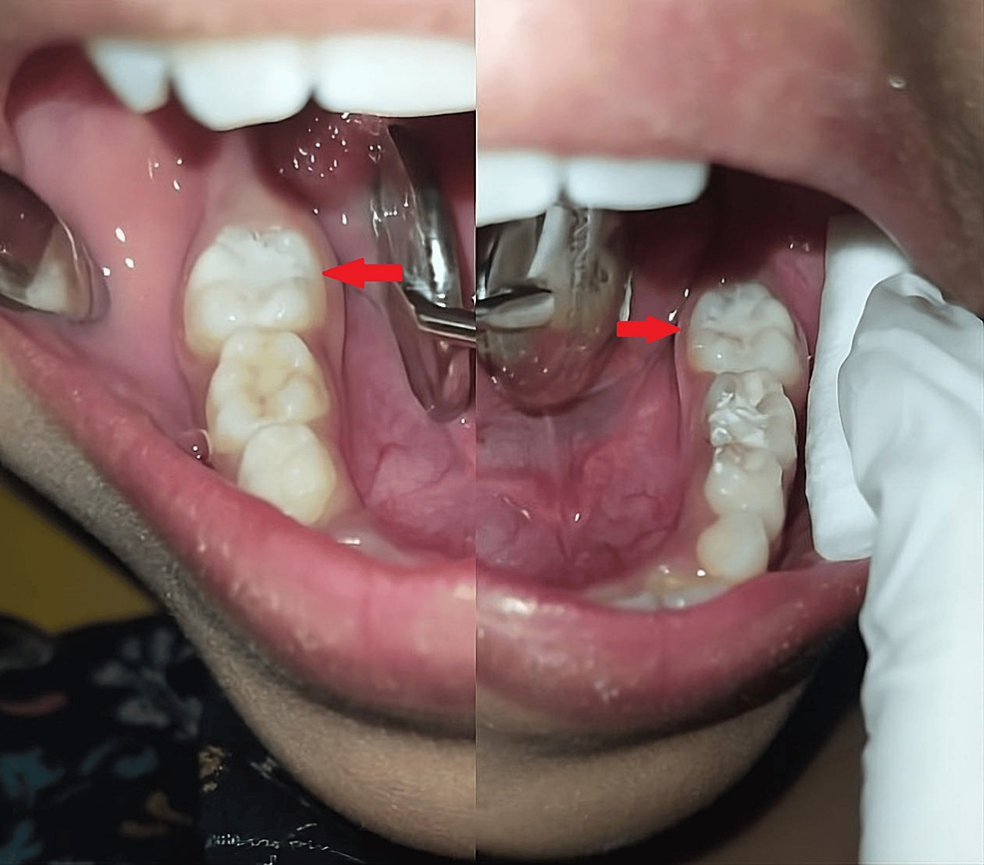
GIC applied as pit and fissure sealant in both left and right permanent 1st molars (arrows) of a seven-year-old patient GIC: Glass Ionomer Cement

After obtaining parental consent, tooth surfaces were meticulously prepared for sealant application. This process involved prophylactic cleaning using pumice slurry on a bristle brush, followed by rinsing. Cotton roll isolation was employed to ensure the elimination of salivary pellicle and plaque. Subsequently, the tooth surface was thoroughly rinsed and air-dried before the application of 20% polyacrylic acid for 10 seconds, followed by removal using air-water spray. GIC was then meticulously applied to the pits and fissures (Figure 1). Participants were instructed not to consume food for at least 1 hour post-procedure. All procedures were performed by the same researchers to maintain consistency across cases.

Outcome assessment

Sealant retention was evaluated at the end of 12 months. It was categorized as complete if present in all parts of pits and fissures, and as absent if lost.

Data collection and analysis

Data were recorded on a specially designed proforma and analyzed using SPSS version 22.0 (IBM Corp., Armonk, NY, USA). Descriptive statistics, including mean and standard deviation, were computed for age. Qualitative variables such as gender, age groups, and residence (rural/urban) were presented in terms of frequency and percentage. Effect modifiers were assessed through data stratification based on mean age, age groups, gender, and living area. Following stratification, the t-test was applied to determine the impact of these factors on sealant retention, with a p-value ≤0.05 considered statistically significant.

## Results

In this study, the age range of participants spanned from 7 to 12 years, with a mean age of 9.24 ± 1.38 years. The majority of patients, comprising 33 individuals (58.8%), fell within the 7-9 years age bracket. Out of the total 56 patients, 27 (48.2%) were male, while 29 (51.8%) were female, as depicted in Table 1.

**Table 1:** General characteristics of study subjects

Variable		Value
Mean age (Years)		9.23 ± 1.39
Age groups	7-9 years	33 (58.8%)
	10-12 years	23 (41.2%)
Gender	Male	27 (48.2%)
	Female	29 (51.8%)
Place of living	Urban	33 (58.8%)
	Rural	23 (41.2%)

Upon assessing the retention of sealants after 12 months, it was found that 31 patients retained the sealant, while 25 experienced sealant loss. Within the age group of 7-9 years, complete retention was observed in 19 patients, whereas in the 10-12 years age group, 12 patients exhibited complete retention. In terms of gender distribution, 17 males and 14 females demonstrated complete retention of the sealant. Notably, the urban cohort displayed higher retention compared to their rural counterparts. The statistical association with age groups and place of living was found to be non-significant. Only the gender was associated significantly with sealant retention (Table 2).

**Table 2:** Comparison of sealant retention with age groups, gender and living area (n=56)

Groups		Retention		p-value
		Yes	No	
Age (years)	7-9	19	14	0.12
	10-12	12	11	
Gender	Male	17	10	0.04
	Female	14	15	
Place of Living	Rural	13	10	0.15
	Urban	18	15	

## Discussion

Dental sealants have proven effective in preventing pit and fissure caries, with recent advancements in fluoride-containing sealants further enhancing their caries-preventive efficacy. Acid conditioning of the enamel prior to sealant application facilitates micromechanical retention of the coating [15,16]. Visual tactile assessment is commonly used to clinically evaluate pit and fissure sealant performance, categorizing sealants as intact, partially lost, or complete lost. In this study, the efficacy of GIC, measured by the retention rate at 12 months, was noted in 31 (55.36%) patients. Another study reported that the efficacy, in terms of complete retention at 12 months, was 52.30% for glass ionomer sealant [17].

Another notable advantage of using glass ionomers as sealants is the reduced working time, as acid etching is unnecessary for achieving chemical bonding to the tooth. This time-saving aspect is particularly crucial for patients with disabilities, where treatment can be challenging, often necessitating physical restraints [18]. Guler and Yilmaz discovered higher retention rates after six months for glass ionomer sealants, with a rate of 82% [19]. In contrast to the short-term effects of topically applied fluoride on dental enamel, glass ionomer sealants initiate a spreading mechanism. This mechanism involves mouth fluid anions being attracted by opposite charges, facilitating an exchange with fluoride, which is then spread to the surface and released. Such sustained release mechanisms ensure optimal physical properties and fluoride release, spanning from days to years. This extended release not only decreases the incidence of caries following acid attacks by up to 35% but also minimizes demineralization to within a few millimeters of the material [20].

Initial observations suggest that High-viscosity Glass Ionomer Cement (HVGIC) exhibits a superior retention rate compared to low-viscosity GIC-based sealants, especially when applied using the press-finger technique. Additionally, studies have shown minimal development of carious lesions on teeth sealed with HVGIC, with no variability dependent on the operator [21]. Current research indicates that loss of retention in GIC sealants primarily occurs due to cohesive failure, where the material fractures but leaves an adhesive layer on the adherent surface. Unlike Composite Resin (CR) sealants, which tend to undergo adhesive failure, leaving fissures exposed and unprotected, GIC sealants maintain their caries preventive effect. This effect is attributed to continuous fluoride leaching and the barrier they create against bacterial invasion, particularly in deeper pits and fissures [22,23].

This study, while rigorously conducted, has several limitations that warrant consideration. Its single-center design may restrict the generalizability of findings, while the relatively small sample size and convenience sampling method could introduce selection bias. The short 12-month follow-up period may not fully capture long-term outcomes. Additionally, the exclusion criteria may limit applicability to certain populations, and the binary assessment of sealant retention oversimplifies potential outcomes.

## Conclusions

Indeed, GIC presents itself as a highly favorable option for sealing pits and fissures due to its commendable retention rates. Its attributes such as being less technique-sensitive, cost-effective, fluoride release and exhibiting good retention make it an excellent choice for pit and fissure sealants. GIC's ability to provide effective sealing while offering ease of application and favorable economic considerations further solidify its status as an outstanding material for this purpose.
